# Machine-learning method for localization of cerebral white matter hyperintensities in healthy adults based on retinal images

**DOI:** 10.1093/braincomms/fcab124

**Published:** 2021-06-03

**Authors:** Benny Zee, Yanny Wong, Jack Lee, Yuhua Fan, Jinsheng Zeng, Bonnie Lam, Adrian Wong, Lin Shi, Allen Lee, Chloe Kwok, Maria Lai, Vincent Mok, Alexander Lau

**Affiliations:** 1 Centre for Clinical Research and Biostatistics, Jockey Club School of Public Health and Primary Care, Faculty of Medicine, The Chinese University of Hong Kong, Shatin, Hong Kong, China; 2 Clinical Trials and Biostatistics Lab, CUHK Shenzhen Research Institute, Shenzhen, China; 3 Margaret KL Cheung Research Centre for Management of Parkinsonism, Therese Pei Fong Chow Research Centre for Prevention of Dementia and Gerald Choa Neuroscience Centre, Faculty of Medicine, The Chinese University of Hong Kong, Shatin, Hong Kong, China; 4 Division of Neurology, Department of Medicine and Therapeutics, Faculty of Medicine, The Chinese University of Hong Kong, Shatin, Hong Kong, China; 5 Department of Neurology, First Affiliated Hospital of Sun Yat-Sen University, Guangdong, China; 6 Key Laboratory for Diagnosis and Treatment of Major Neurological Diseases, National Key Clinical Department, National Key Discipline, Guangzhou 510080, China; 7 BrainNow Research Institute, Shenzhen, Guangdong Province, China; 8 Department of Imaging and Interventional Radiology, The Chinese University of Hong Kong, Shatin, Hong Kong, China; 9 Department of Psychiatry, Faculty of Medicine, The Chinese University of Hong Kong, Shatin, Hong Kong, China

**Keywords:** cerebral small vessel disease, artificial intelligence, stroke, vascular dementia, Alzheimer's disease

## Abstract

Retinal vessels are known to be associated with various cardiovascular and cerebrovascular disease outcomes. Recent research has shown significant correlations between retinal characteristics and the presence of cerebral small vessel disease as measured by white matter hyperintensities from cerebral magnetic resonance imaging. Early detection of age-related white matter changes using retinal images is potentially helpful for population screening and allow early behavioural and lifestyle intervention. This study investigates the ability of the machine-learning method for the localization of brain white matter hyperintensities. All subjects were age 65 or above without any history of stroke and dementia and recruited from local community centres and community networks. Subjects with known retinal disease or disease influencing vessel structure in colour retina images were excluded. All subjects received MRI on the brain, and age-related white matter changes grading was determined from MRI as the primary endpoint. The presence of age-related white matter changes on each of the six brain regions was also studied. Retinal images were captured using a fundus camera, and the analysis was done based on a machine-learning approach. A total of 240 subjects are included in the study. The analysis of various brain regions included the left and right sides of frontal lobes, parietal–occipital lobes and basal ganglia. Our results suggested that data from both eyes are essential for detecting age-related white matter changes in the brain regions, but the retinal parameters useful for estimation of the probability of age-related white matter changes in each of the brain regions may differ for different locations. Using a classification and regression tree approach, we also found that at least three significant heterogeneous subgroups of subjects were identified to be essential for the localization of age-related white matter changes. Namely those with age-related white matter changes in the right frontal lobe, those without age-related white matter changes in the right frontal lobe but with age-related white matter changes in the left parietal–occipital lobe, and the rest of the subjects. Outcomes such as risks of severe grading of age-related white matter changes and the proportion of hypertension were significantly related to these subgroups. Our study showed that automatic retinal image analysis is a convenient and non-invasive screening tool for detecting age-related white matter changes and cerebral small vessel disease with good overall performance. The localization analysis for various brain regions shows that the classification models on each of the six brain regions can be done, and it opens up potential future clinical application.

## Teaser

This study showed that automatic retinal imaging analysis is a convenient, cost-effective and non-invasive screening method to identify and localize cerebral white matter hyperintensities. Future research can correlate the presence of white matter hyperintensities in specific brain regions with various neurological conditions.

## Graphical Abstract

**Graphical Abstract:**
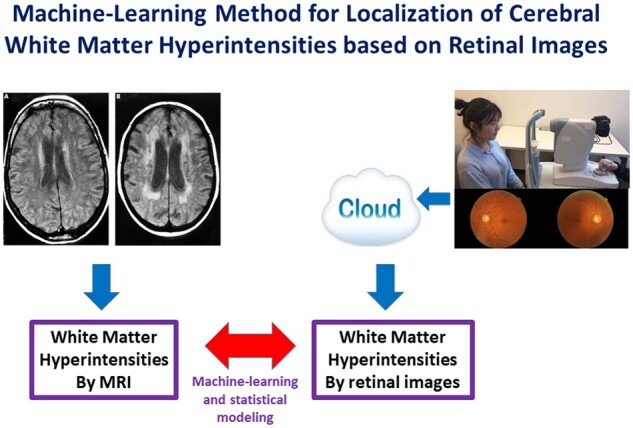

## Introduction

Dementia is a global health problem with an annual societal cost of US$818 billion, affecting 50 million people worldwide, and the number is expected to triple by 2050. The World Health Organization has recently announced a new set of guidelines on primary prevention of dementia through risk factors modification. Still, the crux of the problem remains to be how to identify individuals at risk.^1^ It has been well established that cerebral small vessel disease (SVD) is strongly associated with all-cause dementia, stroke, depression, Parkinsonism and mortality.^2–6^ Cerebral SVD is used to describe a range of pathologies with various aetiologies associated with the small vessels, typically small arteries and arterioles, in the brain.^3^^,^^7^ The manifestation of cerebral SVD can be myriad—the most common findings on brain imaging are white matter hyperintensity (WMH) of presumed vascular origin, lacune of presumed vascular origin, perivascular space and cerebral microbleeds.^7^^,^^8^ In particular, WMH are vigorously studied in recent years.

White matter hyperintensity reflects demyelination and axonal loss in white matter, presumably secondary to chronic ischaemia. It is a highly prevalent phenomenon^9^ related to age and vascular risk factors.^10–12^ WMH implies a 2- to 3-fold risk of incident cognitive decline or dementia and a 3-fold risk of stroke.^3^^,^^13^ Moreover, increasing progression in WMH is a good predictor of these adverse clinical outcomes.^14–16^ Nonetheless, its progression can be curbed or even reversed through vascular risk factors modification and ameliorating the progression is associated with a decreased likelihood of these adverse outcomes.^8^^,^^16^^,^^17^ Since WMH commonly harbours in the brain for years before clinical symptoms appear, this subclinical phase, if identified early with a screening tool, provides a window for risk factors modification.^18^

The current gold standard for assessing WMH remains to be MRI, while other imaging modalities, such as diffusion tensor imaging to detect early white matter changes, are also under development.^11^ However, they are unlikely candidates for population-based screening tool due to limitation by cost, availability and operators’ expertise.

Deep learning (DL) is a family of machine learning methods that has gained considerable attention in the scientific community, especially in medical image processing. DL differs from conventional machine learning methods by its ability to learn the raw data's optimal representation through consecutive nonlinear transformations, achieving increasingly higher levels of abstraction and complexity. DL is based on vast neural networks, and so is its capacity to learn. However, reliable methods must be developed, which take into account the unique features of the images. Indeed, medical images capture patients' anatomy and physiology through the measurements of the geometrical, biophysical, and biochemical properties of their living tissues. These images are acquired with algorithms that exploit complex medical imaging processes whose principles must be well understood in order to be helpful in a specific application. The analysis of the retinal image for detection of WMH is a perfect example.

As retinal and cerebral vessels share the same embryonic origin, the two microvascular systems have similar anatomical and pathophysiological characteristics. It is also well established that retinal vessel architecture correlates well with MRI measures of WMH.^19^ Being the only microvasculature that can be directly seen in our body, we have shown that retinal fundus imaging is an excellent potential candidate for an accurate, rapid, economical, convenient and non-invasive screening tool for WMH and cerebral SVD.^20^ In addition to the estimation of total volume or overall severity grading of WMH, the localization of WMH in various regions of the brain may contain additional valuable information for further understanding of progression or future development of a preventive intervention. In this study, we hypothesized that retinal image analysis could be used to identify the locations of WMH in various brain regions and study the patterns of WMH development.

## Methods

### Study population

The study population was a community-based cohort—The Chinese University of Hong Kong—Risk index for Subclinical Brain Lesions in Hong Kong. The inclusion and exclusion criteria of this cohort have been previously detailed in Lau et al.^20^ In brief, we included participants aged 65 or above who provided written consent and participated in cognitive testing. But we excluded subjects with conditions that would affect the quality of MRI or retinal imaging obtained and those with documented neurological diseases, such as stroke, transient ischaemic attack, dementia and brain tumours. Written informed consents were obtained from all subjects, and the project was done according to the guidelines of the Declaration of Helsinki and approved by the Joint Chinese University of Hong Kong—New Territories East Cluster Clinical Research Ethics Committee (CREC Ref. No. 2012.085).^20^

### Vascular risk factors

Vascular risk factors of the subjects were also considered in this study. Details of the definitions were also described in Lau et al..^20^ Hypertension, diabetes and hyperlipidaemia were defined according to established local guidelines or on current medical treatment. Cardiovascular conditions, such as ischaemic heart disease, arrhythmia and structural heart diseases, were also taken into account in this study.

### Brain MRI acquisition and analysis

Brain MRIs of the subjects were acquired on a 3.0-T scanner using standard protocols as previously reported in Lau et al. 2019: ‘Sequences used in this study include T_1_-weighted, T_2_-weighted and FLAIR. WMH was determined as ill-defined hyperintensities in white matter on FLAIR and T_2_-weighted sequences, but isointense or hypointense with normal brain parenchyma on T_1_-weighed images.^7^ All scans were rated visually by trained independent raters according to the age-related white matter changes (ARWMC) scale.^21^ A global ARWMC score (i.e. highest ARWMC score among the ten brain regions with score 0, 1, 2 and 3 representing nil, focal lesion, early confluent and confluent lesions, respectively) were calculated. Subject with severe or high-risk WMH was defined as having a global ARWMC score ≥ 2. ARWMC score was graded by two independent raters (one neurologist and one trained research assistant) using anonymized MRI data. There was high interrater reliability, and the intraclass correlation coefficient between the raters for the ARWMC global score was 0.909. On top of visual rating, WMH volume was also quantified by a fully automated programme—AccuBrain (BrainNow Medical Technology Limited, Hong Kong, China).’^20^

### Retinal imaging acquisition and analysis

Canon non-mydriatic fundus camera (CR-2 AF, Canon Singapore) and Topcon non-mydriatic retinal camera (TRC-NW6S, Tokyo Optical Co, Tokyo) were used to capture the colour retinal images using a 45° field of view centred on the fovea. Retinal characteristics estimated by the automatic retinal image analysis (ARIA) will be used in the analysis, they include central retinal artery equivalent, central retinal vein equivalent, arteriole–venule ratio calculated as the ratio of central retinal artery equivalent to central retinal vein equivalent, arteriole-venous nipping, arteriole occlusions, presence of haemorrhage and exudates, tortuosity, arterioles and venules bifurcation coefficients, arterioles and venules bifurcation angles, arterioles, venules asymmetry and other variables, such as fractal dimensions, textual parameters will also be used for model building. Since the retinal images were generally of high quality, we have included all retinal images in the analysis after a visual examination according to our standard protocol for quality control.

### Statistical analysis

We used a fully ARIA method to acquire and analyze retinal images in our study. ARIA was applied and validated in different disease cohorts, including stroke, diabetes and chronic kidney disease.^22–24^ The fully ARIA was developed using R and Matlab computer software.^25^^,^^26^ The detailed ARIA method have been reported (US Patent 8787638 B2; http://www.google.com/patents/US8787638). The methods include the use of fractal analysis, high order spectra analysis and statistical texture analysis incorporating a machine learning approach. These approaches were used to estimate the probability of ARWMC score ≥ 2. For the overall validation of the risk of ARWMC, we use a completely separate set of subjects with MRI not previously used in the model building process. A box-plot for the probability of ARWMC score ≥ 2 for the testing samples between ARWMC < 2 (i.e. low-risk group) and ARWMC ≥ 2 (i.e. high-risk group) is shown.

**Figure 1:**
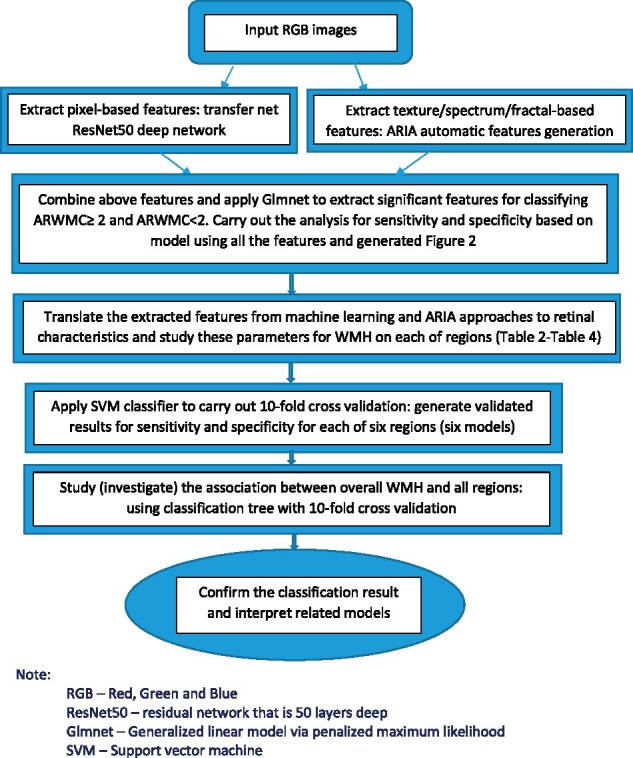
Flowchart for the development of classification model.

For the localization analysis of brain WMH, we applied transfer learning with the pre-trained deep convolutional neural networks ResNet50 to extract features from retinal images.^27^ We then incorporated the extracted image features with the retinal characteristics and estimated the probability of ARWMC scores corresponding to each brain region. The following are a description of the detailed analysis using Matlab. The methodology is shown in the flow chart (Fig. 1). We applied a DL approach such as transfer net of ResNet50 convolutional neural network—input retinal images (RGB and size 224×224×3). Labels are WMH present/absent on each of six regions. The purpose is to generate features based on pixels associated with WMH. Next, we extracted the texture/fractal/spectrum-related features such as high order spectrum and fractal dimensions from our previous ARIA automatic algorithm model. Input retinal images (RGB and size 576×720×3). The purpose is to generate features based on the above three descriptors associated with WMH for each of six regions, respectively. After we extracted the pixel-based features from the above ResNet50 net at the layer of ‘fc1000_softmax’, we combined them with the above extracted features. All of these features will be refined by using the glmnet approach to select important potential features based on penalized maximum likelihood. These refined features were highly associated with WMH for each of the six regions. We then used the above features to estimate retinal characteristics that are meaningful and interpretable for our study (Random forest in Matlab was used). Then we applied a conventional statistical approach such as logistic regression to find the statistically significant risk factors (which is highly associated with WMH for each of six regions).

Finally, we applied the classification and regression tree method to investigate the overall patterns of localization for the presence of WMH for all six brain regions.^28^ The classification and regression tree split the data according to WMH on a specific location of the brain region as a node and applied the splitting criterion to decide the brain region's choice at that particular level. The splitting criterion is the maximum difference in probability estimate for the presence or absence of WMH in that node. The results of the classification and regression tree model were validated using a 10-folder cross-validation method.

### Sample size estimation

We have included 180 subjects from the previous data pool as training samples. From the previous publication, we observed sensitivity and specificity of 93% and 98%, respectively.^20^ Therefore, we postulated that the prospective testing samples' sensitivity and specificity should reach about 90%. To obtain sensitivity and specificity values of 0.9 or higher with a lower bound of at least 0.7 based on 95% confidence intervals (CIs), we need to have at least 25 subjects in each of the ‘high-risk’ group (i.e. global ARWMC ≥ 2) and the ‘low-risk’ group (i.e. ARWMC < 2).^29^ The analysis was carried out once we have acquired an adequate number of cases for subjects with a global ARWMC score ≥ 2. In summary, we prospectively recruited 60 subjects, with 31 subjects in the ‘low-risk’ group and 29 subjects in the ‘high-risk’ group. In the localization analysis, all 240 subjects were used to maximize the number of subjects with ARWMC in each of the brain regions.

### Data availability

The data that support the findings of this manuscript are available from the corresponding author, upon reasonable request.

## Results

### Overall results

The overall data set for this study includes a total of 240 subjects. The characteristics for the 180 samples of training data that are significantly different between those with ARWMC grade 0 and 1, (i.e. low-risk group) and ARWMC grade 2 and 3 (i.e. high-risk group) are shown in Table 1. The average age was about 70 years old, with 54 (30%) males. The high-risk group's average age was significantly higher than that of the low-risk group (*P* = 0.005). There were significantly more hypertension subjects in the high-risk group (78.5%) than the low-risk group (53.5%). The prevalence of diabetes (*P* = 0.020) and WMH volume (*P* < 0.001) is also significantly higher in the high-risk group. For the 60 testing samples, the characteristics are shown in Table 2. Only WMH volume is significantly higher in the high-risk group.

**Table 1:** Characteristics of the study participants—training data (*N* = 180)

	ARWMC < 2 (*N* = 101)	ARWMC ≥ 2 (*N* = 79)	*P*-value
Age, median (IQR)^a^, years	69.00 (66.00–69.00)	71.00 (68.00–75.23)	0.005
Education, median (IQR)^a^, years	7 (4–11.25)	9 (6–12)	0.120
Male, *N* (%)^b^	26 (25.7%)	28 (35.4%)	0.138
MoCA < 21, *N* (%)^b^	33 (32.7%)	27 (34.2%)	0.799
Hypertension, *N* (%)^b^	54 (53.5%)	62 (78.5%)	<0.001
Diabetes Mellitus, *N* (%)^b^	10 (9.9%)	18 (22.8%)	0.020
WMH volume, median (IQR)^a^, ml	2.362 (1.501–4.292)	8.743 (4.161–17.066)	<0.001
Log-transformed WMH volume, mean (IQR)^a^	0.860 (0.406–1.456)	2.168 (1.426–2.837)	<0.001
Frontal lobe (left) ≥ 1, *N* (%)^b^	46 (45.5%)	70 (88.6%)	<0.001
Frontal lobe (right) ≥ 1, *N* (%)^b^	50 (49.5%)	70 (88.6%)	<0.001
Parietal–occipital lobe (left) ≥ 1, *N* (%)^b^	30 (29.7%)	64 (81.0%)	<0.001
Parietal–occipital lobe (right) ≥ 1, *N* (%)^b^	28 (27.7%)	66 (83.5%)	<0.001
Basal Ganglia (left) ≥ 1, *N* (%)^b^	3 (3.0%)	20 (25.3%)	<0.001
Basal Ganglia (right) ≥ 1, *N* (%)^b^	5 (5.0%)	21 (26.6%)	<0.001

ARWMC, age-related white matter changes; IQR, interquartile range; MoCA, Montreal Cognitive Assessment; WMH, white matter hyperintensity.

a Mann–Whitney U-test.

b Chi-square test.

**Table 2.** Characteristics of the study participants—testing data (*N* = 60)

	ARWMC < 2 (*N* = 31)	ARWMC ≥ 2 (*N* = 29)	*P*-value
Age, median (IQR)^a^, years	71.44 (69.48–73.51)	71.97 (70.32–78.04)	0.119
Education, median (IQR)^a^, years	11 (4–12)	7 (2.5–11)	0.475
Male, *N* (%)^b^	9 (71.0%)	7 (24.1%)	0.668
MoCA < 21, *N* (%)^b^	7 (22.6%)	10 (34.5%)	0.301
Hypertension, *N* (%)^b^	27 (87.1%)	22 (75.9%)	0.244
Diabetes Mellitus, *N* (%)^b^	10 (32.3%)	8 (27.6%)	0.560
WMH volume, median (IQR)^a^, ml	2.041 (0.786–3.757)	9.654 (4.613–15.349)	<0.001
Log-transformed WMH volume, mean (IQR)^a^	0.712 [(−0.242)–1.322]	2.267 (1.529–2.731)	<0.001
Frontal lobe (left) ≥ 1, *N* (%)^b^	16 (51.6%)	27 (93.1%)	<0.001
Frontal lobe (right) ≥ 1, *N* (%)^b^	14 (45.2%)	28 (96.6%)	<0.001
Parietal–occipital lobe (left) ≥ 1, *N* (%)^b^	12 (38.7%)	26 (89.7%)	<0.001
Parietal–occipital lobe (right) ≥ 1, *N* (%)^b^	12 (38.7%)	27 (93.1%)	<0.001
Basal Ganglia (left) ≥ 1, *N* (%)^b^	7 (22.6%)	15 (51.7%)	0.019
Basal Ganglia (right) ≥ 1, *N* (%)^b^	6 (19.4%)	16 (55.2%)	0.004

ARWMC, age-related white matter changes; IQR, interquartile range; MoCA, Montreal Cognitive Assessment; WMH, white matter hyperintensity.

a Mann–Whitney U-test.

b Chi-square test.

**Figure 2:**
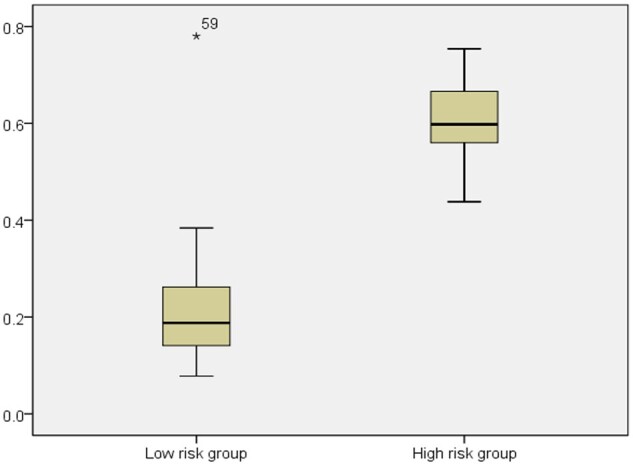
**Probability of ARWMC ≥ 2 estimated from retinal images for the low-risk group (ARWMC < 2, Group = 0) and the high-risk group (ARWMC ≥ 2, Group = 1)**.

The retinal image analysis model on calculating the probability of having ARWMC score ≥ 2 was developed based on the 180 training samples. The model was then validated using the 60 testing samples. Figure 2 shows a box-plot for the probability of ARWMC ≥ 2 in the testing samples between the ARWMC < 2 (i.e. low-risk group) and the ARWMC ≥ 2 (i.e. high-risk group). The median probabilities of the low and high-risk groups were about 0.2 and 0.6, respectively, while the mean probabilities for the low and high-risk groups were 0.218 (*n* = 31, SD = 0.1337, 95% CI from 0.169–0.267) and 0.609 (*n* = 29, SD = 0.0876, 95% CI from 0.575–0.642), respectively. The probabilities of the two groups are significantly different, and the classification result is excellent.

Among the 29 subjects with an ARWMC score ≥ 2, 26 were correctly identified as high risk by the model with a sensitivity of 89.7% (95% CI: 0.715–0.973) using a cutoff point of 0.5. The three incorrect classifications have probabilities only slightly lower than the 0.5 cutoffs. Among the 31 subjects with grades 0 and 1 age-related white matter hyperintensities, 30 were correctly classified as low risk with a specificity of 96.8% (95% CI: 0.815–0.998).

**Figure 3:**
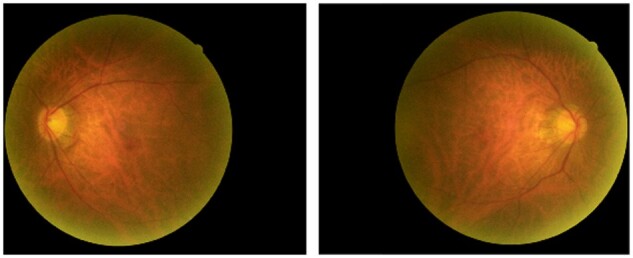
The retinal images with poor quality.

This result demonstrated that the accuracy of the classification is high except for a single case in the group ARWMC grade 1 which is an outlier due to poor retinal image quality, as the retinal vessel’s structure was not clearly presented due to borderline vagueness of background for both retinal images (Fig. 3). The mean probabilities of the four ARWMC groups from grades 0 to 3 were 0.173 (*n* = 4, SD = 0.0864, 95% CI: 0.035–0.310), 0.225 (*n* = 27, SD = 0.1393, 95% CI: 0.170–280), 0.593 (*n* = 19, SD = 0.0840, 95% CI: 0.552–0.633) and 0.639 (*n* = 10, SD = 0.0903, 95% CI: 0.575–0.704) respectively.

### Localization analysis of brain WMH

We first investigated if there are significant retinal parameters significantly associated with WMH in various regions of the brain. All 240 subjects were used in this analysis. This study's brain regions included left and right frontal lobes, parietal–occipital lobes and basal ganglia. The specific parameters that were significantly associated with the ARWMC in the bilateral frontal lobes, parietal–occipital lobes and basal ganglia are listed in Tables 3–5. Only the retinal parameters that were statistically different at a *P*-value of less than 0.20 level between low and high-risk groups are shown in Tables 3–5. These results suggested that data from both eyes are essential for the detection of WMH in the unilateral brain regions, but the retinal parameters useful for estimation of the probability of WMH in each of the brain regions may different for different locations. Classification models used for each of the regions was developed using a similar methodology.

**Table 3:** Univariate analysis of retinal parameters for WMH on frontal lobe

	Frontal lobe—right side						Frontal lobe—left side					
	Left eye			Right eye			Left eye			Right eye		
	Low-risk mean (*n* = 154)	High-risk mean (*n* = 60)	*P*-value	Low-risk mean (*n* = 154)	High-risk mean (*n* = 60)	*P*-value	Low-risk mean (*n* = 154)	High-risk mean (*n* = 60)	*P*-value	Low-risk mean (*n* = 154)	High-risk mean (*n* = 60)	*P*-value
MBCV	1.25	1.24	0.117									
Mvangle	73.13	73.60	0.169				73.14	73.61	0.152			
Tortuosity	0.351	0.375	0.018									
Haemorrhage	0.321	0.339	0.010	0.358	0.370	0.100						
Exudates	0.321	0.338	0.057	0.296	0.308	0.060				0.296	0.309	0.045
Mvasymmetry				0.727	0.732	0.046	0.713	0.710	0.166			

Exudates, estimated exudate (probability); Haemorrhage, estimated haemorrhage (probability); Mvasymmetry, mean of asymmetry index for venules; MBCA, mean of the bifurcation coefficient for arterioles; Mvangle, mean of the bifurcation angles for venules (degree); Tortuosity, estimated artery tortuosity (probability).

**Table 4:** Univariate analysis of retinal parameters for WMH on parietal–occipital lobe

	Parietal–occipital Lobe—Right Side						Parietal–occipital Lobe—Left Side					
	Left Eye			Right Eye			Left Eye			Right Eye		
	Low-risk mean (*n* = 156)	High-risk mean (*n* = 58)	*P*-value	Low-risk mean (*n* = 156)	High-risk mean (*n* = 58)	*P*-value	Low-risk mean (*n* = 156)	High-risk mean (*n* = 58)	*P*-value	Low-risk mean (*n* = 156)	High-risk mean (*n* = 58)	*P*-value
CRVE	18.60	18.46	0.191	18.25	18.01	0.09	18.63	18.42	0.057	18.27	17.98	0.04
MBCV	1.25	1.24	0.168				1.25	1.24	0.087			
Haemorrhage	0.323	0.333	0.172				0.322	0.335	0.067	0.359	0.367	0.190
Mvasymmetry				0.727	0.733	0.014				0.726	0.733	0.004
MBCA										1.66	1.68	0.175

CRVE, central retinal vein equivalent (pixels); Haemorrhage, estimated haemorrhage (probability); MBCV, mean of the bifurcation coefficient for venules; Masymmetry, mean of asymmetry index for arterioles.

**Table 5:** Univariate analysis of retinal parameters for WMH on basal ganglia lobe

	Basal Ganglia Lobe—Right Side						Basal Ganglia Lobe—Left Side					
	Left Eye			Right Eye			Left Eye			Right Eye		
	Low-risk mean (*n* = 195)	High-risk mean (*n* = 19)	*P*-value	Low-risk mean (*n* = 195)	High-risk mean (*n* = 19)	*P*-value	Low-risk mean (*n* = 195)	High-risk mean (*n* = 19)	*P*-value	Low-risk mean (*n* = 195)	High-risk mean (*n* = 19)	*P*-value
CRAE	11.92	11.65	0.024	11.64	11.09	<0.001	11.92	11.72	0.084	11.64	11.18	0.003
CRVE	18.62	17.95	<0.001	18.26	17.31	<0.001	18.61	18.03	<0.001	18.24	17.56	0.001
MBCV	1.25	1.19	<0.001	1.25	1.21	<0.001	1.25	1.20	0.001	1.25	1.22	0.006
Mvasymmetry	0.712	0.717	0.073	0.727	0.739	0.001				0.728	0.734	0.077
Nipping	0.340	0.317	0.103				0.341	0.315	0.064			
Haemorrhage	0.324	0.352	0.097	0.360	0.376	0.152						
MBCA				1.66	1.72	0.143						
Masymmetry				0.843	0.851	0.009						
Tortuosity				0.369	0.391	0.128	0.360	0.329	0.051	0.369	0.387	0.194
Aocclusion				0.107	0.136	0.146				0.107	0.140	0.017
Mvangle										72.64	73.38	0.191
Maangle										71.66	72.97	0.002

Aocclusion, estimated arterioles occlusion (probability); CRAE, central retinal artery equivalent (pixels); CRVE, central retinal vein equivalent (pixels); Haemorrhage, estimated haemorrhage (probability); MBCV, mean of the bifurcation coefficient for venules; MBCA, Mean of the bifurcation coefficient for arterioles; Masymmetry, mean of Asymmetry index for arterioles; Maangle, mean of the bifurcation angles for arterioles (degree); Mvangle, mean of the bifurcation angles for venules (degree); Nipping, estimated arteriole-venous nicking (probability); Tortuosity, estimated artery tortuosity (probability).

**Figure 4:**
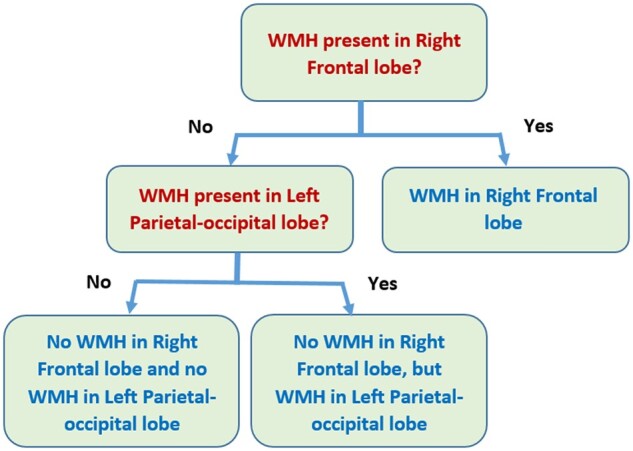
Regression tree results on localization of cerebral white matter hyperintensities.

Since the presence of WMH in each of the brain regions are not entirely independent, we have also analyzed the importance and relationship among the brain regions with respect to the presence of WMH using classification and regression tree analysis. This method takes into account potential interactions among various brain regions. The response variable for the classification and regression tree analysis was ARWMC ≥ 1, i.e. the presence of WMH. An optimal splitting criterion of the brain regions into homogeneous groups was used to determine the best classification results. We found that there are at least three subgroups of subjects identified to be important for the localization of WMH, namely Group 1: those with WMH in the right frontal lobe, Group 2: those without WMH in the right frontal lobe but with WMH in the left parietal–occipital lobe, and Group 3: the rest of subjects without WMH in the right frontal lobe and left parietal–occipital lobe. The results are shown in Fig. 4. WMH of the six brain regions were used to determine the overall severity grading of ARWMC in the form of a classification and regression tree model. The sensitivity and specificity were calculated based on the cross-validation approach with 98.8% and 92.0%, respectively, an area under the receiver operating characteristic curve of 0.955.

We further investigated the overall grading of ARWMC for each of the three subgroups, the mean probabilities of ARWMC ≥ 2 and their 95% CIs for participants in Groups 1, 2 and 3 were 0.563 (95% CI: 0.510, 0.615), 0.191 (95% CI: 0.065, 0.316) and 0.189 (95% CI: 0.151, 0.228), respectively. This result shows that Group 1 is significantly higher than Groups 2 and 3 for the probability of ARWMC ≥ 2. There was also a significant difference among the three subgroups for the proportion of subjects with hypertension, 131/158 (82.9%), 10/12 (83.3%) and 23/37 (62.2%). The three subgroups' differences were significant, with a *P*-value of 0.019 with 2 degrees of freedom chi-square test. Further study on patterns of localization of WMH for various brain regions and their association with the clinical and cognitive outcome may be useful.

## Discussion

Our study showed that ARIA is a good potential candidate to be an accurate, rapid, economical, convenient and non-invasive screening tool for WMH and cerebral SVD with an excellent overall performance of 89.7% sensitivity and 96.8% specificity and 93.3% accuracy in a prospective setting for validation. As mentioned before, earlier detection of WMH prompts risk factors modification before clinical symptoms appear.^18^ Studies have been carried out to determine effective ways to slow the progression of WMH. A long-term randomized trial demonstrated that weight loss and behavioural intervention has a significant effect on reduction in WMH volumes and brain atrophy.^30^ Tighter blood pressure control through pharmacological intervention and lifestyle modification were also associated with a more remarkable fall in WMH volume.^31^ A combination of sartans and low-dose statins was also found to be effective in slowing WMH progression and cognitive decline.^32^ Therefore, with ARIA being an accurate, economical and convenient screening tool for WMH, it allows early primary prevention for advanced diseases, such as dementia and stroke.

Our ARIA model also analyses the localization of brain WMH for all six brain regions by gauging the retinal characteristics. Evidence has highlighted the value in assessing WMH volume in strategic white matter tracts, which correlates better with variance in cognitive functioning than the total WMH volume, healthy and diseased individuals alike.^33–37^ Of note, greater WMH volume in the anterior thalamic radiation and the forceps minor are significantly associated with poor processing speed and executive functioning,^34^^,^^37–39^ WMH volume in the forceps major and minor is found relevant to poor memory^35^^,^^38^^,^^39^; both independent of total WMH volume. Nonetheless, the topography of WMH is still a topic not receiving the attention it deserves owing to the limited availability of advanced lesion-symptom mapping. In this case, the ARIA model has undoubtedly provided a more economical and convenient alternative to the current advanced lesion-symptom analysis. The ability of ARIA to assess strategically located WMH has several implications in digital public health and is a potential tool to improve health equity.

First, understanding regionally located WMH burden might help diagnose and risk prediction of neurodegenerative disorders and the cognitive domains that are more likely to be impaired. For example, multiple studies have shown the association of higher parietal WMH load with an incidence of Alzheimer's disease and its known risk factors.^14^^,^^40^^,^^41^ Moreover, higher WMH burden in frontal and parietal lobes was also found to correlate with increased risk of Parkinsonism.^40^^,^^42^ Precedent post-stroke studies have also established an association of WMH locations with both post-stroke global cognitive impairment and specific cognitive domain deficits.^43–47^ Studies on the relationship between strategically located WMH and cognitive conditions are still ongoing. We are optimistic that more exciting discoveries are coming up. With more associations established, assessing WMH loads in strategic locations will help healthcare professionals identify community-dwelling subjects with a high risk of developing certain neurological conditions. It can also help recognize acute care patients with potential, more unsatisfactory clinical outcome. Primary, secondary or tertiary preventive resources can then be preferentially allocated to these patients in need, achieving health equity.

Second, mounting evidence shows that different neuropsychiatric symptoms (NPS) are associated with different locations of WMH lesions.^48–50^ Given that NPS are ubiquitous in people with neurodegenerative disorders. ARIA might serve as a valuable and quick tool to identify the possible underlying neuropathologies of NPS and predict the prognosis in people who have already presented the symptoms. It might also help predict the risk of developing NPS in those still free of symptoms.

Third, the assessment of WMH accumulation in strategic white matter tracts might facilitate personalised medicine development. Take aerobic exercise training as an example. Systemic reviews and meta-analyses have demonstrated that personalizing the exercise modality may significantly benefit individuals with deficits in specific cognitive subdomains. For example, aerobic exercise improves executive function to a greater extent than resistance training,^51^^,^^52^ while resistance training has more significant memory effects.^42^ Moreover, multimodal training has more pronounced effects on enhancing episodic memory and verbal fluency than aerobic training alone.^51^ On top of cognitive test results, ARIA may provide us additional information on the cognitive profiles of the screened individuals; hence we can allocate them to the modality of exercise classes that can benefit them most, achieving health equity.

We acknowledged that there are several limitations to this study. First, the sample size is limited as it is expensive to have many subjects to have MRI and carry out extensive grading of WMH. Second, the regional WMH load in this group is not as high as in other patient groups. However, this study is essential because it represents a basic norm from an average population and proof of concept study.

For the future direction of the study, we aim to explore the ability of ARIA to differentiate deep and periventricular WMH on top of location. Current evidence demonstrates a stronger association between cognitive declines periventricular than deep WMH.^53^ Moreover, we also aim to study the ability of ARIA to assess the severity and location of other modalities of cerebral SVD, such as lacunar infarcts and cerebral microbleeds.

## Funding

This study was supported by the Hong Kong Innovation and Technology Fund—Midstream Research Programme (MRP/037/17X).

## Competing interest

B.Z. and J.L. are the inventors of patent US8787638 B2 (‘Method and device for retinal image analysis’) licensed to Health View Bioanalytic Limited, received royalties through the Chinese University of Hong Kong. B.Z. and J.L. are founders and shareholders of Health View Bioanalytic Limited, Bioanalytic Holdings Limited, and Bioanalytic International Holdings Limited. ML is a director of Bioanalytic Holdings Limited, and Bioanalytic International Holdings Limited. BZ is co-founder and shareholder of Beth Bioinformatics Company Limited. The remaining authors have no conflict of interest to declare.

## Abbreviations

ARIA =: automatic retinal image analysis
ARWMC =: age-related white matter changes
CI =: confidence interval
CRVE =: central retinal vein equivalent
DL =: deep learning
NPS =: Neuropsychiatric symptoms
SD =: standard deviation
SVD =: small vessel disease
WMH =: white matter hyperintensity
